# Evaluation of *CNTNAP2* gene polymorphisms for exfoliation syndrome in Japanese

**Published:** 2012-05-31

**Authors:** Ai Shimizu, Yoshimasa Takano, Dong Shi, Shunji Yokokura, Yu Yokoyama, Xiaodong Zheng, Atsushi Shiraishi, Yuichi Ohashi, Toru Nakazawa, Nobuo Fuse

**Affiliations:** 1 Department of Ophthalmology, Tohoku University Graduate School of Medicine, Sendai, Japan; 2 Department of Ophthalmology, the Fourth Affiliated Hospital, China Medical University, Shenyang, China; 3 Department of Ophthalmology, Ehime University School of Medicine, Ehime, Japan; 4 Department of Integrative Genomics, Tohoku Medical Megabank Organization (ToMMo), Tohoku University, Sendai, Japan

## Abstract

**Purpose:**

To investigate the contactin-associated protein-like 2 (*CNTNAP2*) gene for single-nucleotide polymorphisms (SNPs) in Japanese patients with the exfoliation syndrome (XFS).

**Methods:**

One hundred and eight unrelated Japanese patients with the XFS, and 199 normal controls were studied. Genomic DNA was extracted from the leukocytes of the peripheral blood, and 8 SNPs, rs826802, rs1404699, rs7803992, rs700308, rs4725736, rs2107856, rs2141388, and rs6970064, were amplified by polymerase chain reaction (PCR), directly sequenced, and genotyped.

**Results:**

The allele frequencies of rs1404699 (p=8.57XE-3, odds ratio (OR)=1.59, 95% confidential intervals (CI); 1,12–2.24) and rs7803992 (p*=*5.43XE-4, OR=1.86, 95% CI; 1.31–2.65) were statistically significantly different between XFS and controls. In addition, there were significant differences in these genotype frequencies (p=0.0197 and 1.75XE-3). The allele and the genotype frequencies of rs2107856 and rs2141388, which were statistically significant SNPs in an earlier study, were not significantly different.

**Conclusions:**

The variants, rs1404699 and rs7803992, of *CNTNAP2* should be associated with XFS in the Japanese population.

## Introduction

The exfoliation syndrome (XFS; OMIM 177650) is a generalized disorder of the extracellular matrix and is characterized clinically by the pathological accumulation of abnormal fibrillar material in the anterior segment of the eye [1-3]. This predisposes the eye to glaucomatous optic neuropathy. The XFS has also been associated with lens zonule weakness, severe chronic secondary open-angle glaucoma, cataract formation, and also a spectrum of other serious spontaneous and surgical intraocular complications.

The prevalence of XFS varies markedly between populations being highest in Scandinavian countries, while the Anglo-Celtic Caucasians have a markedly lower prevalence [4-7]. The incidence increases with age and is highest in the age group between 70 and 80 years [5]. The prevalence of XFS in Japan was reported to be 1.1% in one study [8] and 4.8% in another study [9].

Thorleifsson et al. [10] found a strong association between single-nucleotide polymorphisms (SNPs) in the lysyl oxidase–like 1 (*LOXL1*) gene and XFS in the Swedish and Icelandic populations using a genome-wide association study (GWAS). This association was replicated in the United States of America [11-13] and also in other populations [14-23].

*LOXL1* is a member of the lysyl oxidase family of proteins that catalyzes the oxidative deamination of lysine residues of tropoelastin [24]. The homeostasis of elastic fibers requires the lysyl oxidase-like 1 protein [25], and *LOXL1* plays an important role in elastogenesis. Thus, it is quite possible that defects in *LOXL1* can cause features of XFS that result from an aberrant production of elastin and accumulation of fibrillar materials in the anterior segment of the eye.

A GWAS was recently performed using a DNA-pooling approach, and a single genotype at the contactin-associated protein-like 2 (*CNTNAP2*) locus had significant associations between XFS and exfoliation glaucoma and two SNPs (rs2107856 and rs2141388). These findings were confirmed in an independent German cohort but not in an Italian cohort [26]. *CNTNAP2* is a large gene spanning 2.3 mb of DNA on chromosome 7 and has 24 exons, and codes for the contactin-associated protein-like 2 (*CNTNAP2,* also called Caspr2). CNTNAP2 is member of the neurexin superfamily [27,28] and is possibly involved in stabilizing the location of the potassium channels in the juxtaparanodal region of the neuron [27]. It has been suggested to be a candidate gene for various neuropsychiatric disorders, e.g., the cortical dysplasia-focal epilepsy syndrome [29] and Pitt-Hopkins-like mental retardation [30]. However, its exact function and regulation are not known.

The purpose of this study was to investigate 8 SNPs variations in *CNTNAP2* in Japanese patients with the XFS.

## Methods

One hundred and eight unrelated Japanese patients with XFS (mean age 73.61±6.75 years; 57 men, 51 women) and 199 controls (mean age 69.7±11.3 years; 101 men, 98 women) were studied. The controls were matched by age and gender. The XFS group included 85 exfoliation glaucoma (XFG) patients. They were examined at the ophthalmic clinic of the Tohoku University Hospital, Sendai, Japan, and the Ehime University Hospital, Ehime, Japan. The purpose and procedures were explained to all patients, and an informed consent was obtained. This study was approved by the Institutional Review Boards of the Tohoku University and Ehime University, and the procedures used conformed to the tenets of the Declaration of Helsinki.

Routine ophthalmic examinations were performed on all patients. The criteria used to classify a patient as having XFS was an open anterior chamber angle with accumulation of abnormal fibrillar material in the anterior segment of the eye. In addition, three other criteria for XFG had to be met: 1) applanation intraocular pressure (IOP) >22 mmHg in each eye; 2) glaucomatous cupping in each eye including a cup-to-disc ratio >0.7; and 3) visual field defects determined by Goldmann perimetry and/or Humphrey field analyzer consistent with the glaucomatous cupping in at least one eye. The control subjects had the following characteristics: 1) IOP less than 22 mmHg; 2) normal optic discs; and 3) no family history of glaucoma.

**Figure 1:**
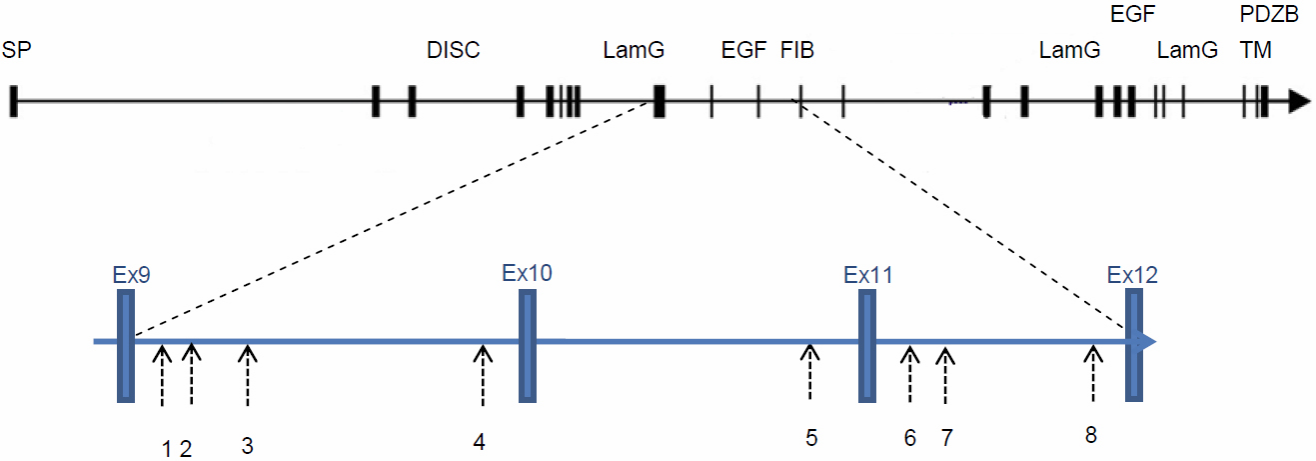
*CNTNAP2* gene structure. The 8 SNPs studied were; 1. rs826802, 2. rs144699, 3. rs7803992, 4. rs700308, 5. rs4725736, 6. rs2107856, 7. rs2141388, and 8. rs6970064. SP, signal peptide; DISC, discoidin-like domain; LamG, laminin-G domain; EGF, epidermal growth factor like domain; FIB, fibrinogen-like domain;TM, transmembrane region; PDZBD, PDZ-domain binding site.

Genomic DNA was extracted from the leukocytes of peripheral blood and purified with the Qiagen QIAamp Blood Kit (Qiagen, Valencia, CA). Genomic DNA was extracted from the leukocytes of the peripheral blood, and the 6 SNPs, rs1404699, rs700308, rs4725736, rs2107856, rs2141388, and rs6970064, were chosen from the earlier studies. Two newly identified SNPs, rs826802 and rs7803992, were designed around intron 9 of the gene. The *CNTNAP2* gene structure with the location of the 8 SNPs is shown in Figure 1. They were amplified by polymerase chain reaction (PCR), directly sequenced, and genotyped. The amplifications were performed at 60 °C annealing temperature. The PCR fragments were purified with ExoSAP-IT (USB, Cleveland, OH), sequenced by the BigDye^TM^ Terminator v3.1 Cycle Sequencing Kit (Perkin-Elmer, Foster City, CA) by an automated DNA sequencer (ABI PRISM^TM^ 3100 Genetic Analyzer, Perkin-Elmer). The allele frequencies, genotypes, and haplotypes of the *CNTNAP2* SNPs were determined.

### Statistical analysis

The significance of associations between the phenotype and SNPs were determined by contingency table analysis using chi-square or Fisher's exact test. The odds ratios, approximating to relative risks, were calculated as a measure of the association between the *CNTNAP2* allele frequency and the phenotype. For each odds ratio, the 95% confidence intervals were calculated. The inferred haplotypes, quantified between all pairs of biallelic loci, were estimated using the SNPAlyze program version 7.0 (Dynacom, Yokohama, Japan). Additionally, a permutation test was performed to test the deviations of allelic frequencies of the SNPs and haplotypes. The Hardy–Weinberg equilibrium was analyzed using gene frequencies obtained by simple gene counting and the chi-square test with Yates' correction for comparing observed and expected values.

## Results

The allele frequencies and genotypes of the 8 *CNTNAP2* SNPs, rs826802, rs1404699, rs7803992, rs700308, rs4725736, rs2107856, rs2141388, and rs6970064, were determined in the XFS patients.

### Distribution of *CNTNAP2* variants in XFS patients and control subjects

The allele frequencies of rs1404699 (p=8.57XE-3, odd ratio (OR)=1.59, 95% confidential intervals (CI); 1,12–2.24) and rs7803992 (p=5.43XE-4, OR=1.86, 95% CI; 1.31–2.65) were statistically significant between the XFS group and the control group (Table 1). There were also significant differences in these genotype frequencies (p=0.0197 and 1.75XE-3; Table 2). Only the rs7803992 was significantly different between the XFG group and the control group (p=0.016; Table 1). Compared with the allele frequencies of rs2107856 and rs2141388 statistically significant SNPs in a previous study [26], our results showed no significantly difference between the XFS group and the control group (Table 1). Also, the genotype frequencies of those in *CNTNAP2* were not significantly higher in the two groups than in the control group (Table 2).

**Table 1:** *CNTNAP2* allele frequencies in patients with exfoliation syndrome and in controls in Japanese.

		**MAF in this study**			MAF in previous study*		
**dbSNP**	**Allele**	**XFS (n=108) XFG (n=85)**	**Control (n=199)**	**p-value**	**XFS (n=770)**	**Control (n=444)**	**p-value**
rs826802	T	0.435	0.372	0.0884	N/A	N/A	N/A
		0.429		0.0198			
rs1404699	T	0.412	0.307	8.57XE-3	0.445	0.397	0.0225
		0.388		0.0581			
rs7803992	G	0.394	0.259	5.43XE-4	N/A	N/A	N/A
		0.359		0.016			
rs700308	A	0.407	0.432	0.553	0.138	0.103	0.0117
		0.412		0.653			
rs4725736	A^1^	0.472	0.402	0.093	0.585	0.637	0.0121
		0.441		0.385			
rs2107856	G^2^	0.450	0.432	0.687	0.709	0.776	0.0003
		0.441		0.843			
rs2141388	C^3^	0.444	0.437	0.863	0.709	0.777	0.0002
		0.441		0.930			
rs6970064	A^4^	0.181	0.123	0.0524	0.418	0.463	0.0306
		0.182		0.0631			

*reported by Krumbiegel et al. [26]. MAF; minor allele frequency, XFS; Exfoliation Syndrome, XFG; Exfoliation Glaucoma. The significance of the association was determined by a contingency table analysis using the χ^2^ test. Upper columns show XFS data, and lower columns show XFG data. 1. There was a difference between the Caucasian and Japanese. Minor allele in previous study was C. 2. Minor Allele in previous study was T. 3. Minor Allele in previous study was T. 4. Minor Allele in previous study was G.

**Table 2:** Frequency of genotypes *CNTNAP2* gene in patients with exfoliation syndrome and in controls in Japanese.

**dbSNP**	**Allele**	**XFG (n=108)**	p value*	**XFG (n=85)**	p value*	**Control (n=199)**
rs826802	G/G	36 (33.3)	0.224	27 (31.8)	0.425	77 (38.7)
	G/T	50 (46.3)		43 (50.6)		96 (48.2)
	T/T	22 (20.4)		15 (17.6)		26 (13.1)
rs1404699	C/C	38 (35.2)	0.0197	32 (37.6)	0.121	93 (46.7)
	C/T	51 (47.2)		40 (47.1)		90 (45.2)
	T/T	19 (17.6)		13 (15.3)		16 (8.1)
rs7803992	A/A	38 (35.2)	1.75XE-3	31 (36.5)	6.22XE-3	112 (56.3)
	A/G	55 (50.9)		47 (55.3)		71 (35.7)
	G/G	15 (13.9)		7 (8.2)		16 (8.0)
rs700308	G/G	45 (41.7)	0.0402	33 (38.8)	0.282	63 (31.7)
	G/A	38 (35.2)		34 (40.0)		100 (50.3)
	A/A	25 (23.1)		18 (21.2)		36 (18.1)
rs4725736	C/C	34 (31.5)	0.0659	27 (31.8)	0.385	69 (34.7)
	C/A	46 (42.6)		41 (48.2)		100 (50.3)
	A/A	28 (25.9)		17 (20.0)		30 (15.1)
rs2107856	T/T	39 (36.1)	0.091	29 (34.1)	0.541	63 (31.7)
	T/G	41 (38.0)		37 (43.5)		100 (50.3)
	G/G	28 (25.9)		19 (22.4)		36 (18.1)
rs2141388	T/T	39 (36.1)	0.100	29 (34.1)	0.470	61 (30.7)
	T/C	42 (38.9)		37 (43.5)		106 (53.3)
	C/C	27 (25.0)		19 (22.4)		32 (16.1)
rs6970064	G/G	74 (68.5)	0.0315	58 (68.2)	0.0345	151 (75.9)
	G/A	29 (26.9)		23 (27.1)		47 (23.6)
	A/A	5 (4.6)		4 (4.7)		1 (0.5)

XFS; Exfoliation Syndrome, XFG; Exfoliation Glaucoma. Data presented are number of patients, unless otherwise indicated. The significance of the association was determined by a contingency table analysis using the χ^2^ test.

The genotype frequencies of rs700308 and rs6970064 were statistically significant (p=0.0402 and 0.0315), but the allele frequencies were not significantly different (p=0.553 and 0.0524) between the XFS group and the control group. All SNPs adhered to the Hardy–Weinberg expectations (p>0.05).

### Haplotype analyses at *CNTNAP2* LD block in the Japanese population

The inferred haplotypes between all pairs of biallelic loci on rs1404699 and rs7803992 were estimated (Table 3). The haplotype-based associations were tested with a 1,000 iterated permutation test. Four major haplotypes; C-A, T-G, T-A, C-G (each frequency >5%) were found in the XFS subjects and normal controls. T-G was over-represented in the XFS subjects with a highly significant difference in frequency compared to the control group (0.327 versus 0.202; p=0.003). In addition, the C-A haplotype was significantly less represented in the XFS subjects (0.522 versus 0.637; p=0.003).

**Table 3:** Haplotype analysis with rs1404699 and rs7803992 in patients with exfoliation syndrome and in controls in Japanese.

**Haplotype**	**Overall**	**XFS**	**Control**	**p-value**
C-A	0.5966	0.5217	0.637	0.003
T-G	0.2464	0.3273	0.2024	0.003
T-A	0.0972	0.0847	0.1041	0.489
C-G	0.0597	0.0662	0.0564	0.708

XFS; Exfoliation Syndrome. The significance of the association was determined by a contingency table analysis using the χ^2^ test.

### Two locus analyses

A strong correlation between variants in *LOXL1* and XFS has been reported [10], *LOXL1* common risk haplotype is T-G (the major alleles T of the coding SNPs rs1048661 and major alleles G of the coding SNPs rs3825942) in Japan, instead of G-G in Europeans. We investigated how the variants in *LOXL1* gene were related to *CNTNAP2*. We sorted our subjects for carriers and non-carriers of the risk haplotype T-G (Table 4). The numbers in the subgroup of non-T-G carriers was quite small, and there was no association of *CNTNAP2* SNPs with the *LOXL1* non-risk haplotype (Table 4; p=0.53 and 0.69, respectively). Besides the subgroups risk of T-G carriers, there was no significant association (Table 4; p=0.072 and 0.084, respectively).

**Table 4:** Association of *LOXL1* common-risk haplotype T-G, composed of rs1048661 and rs3825942, with *CNTNAP2* SNPs rs1404699 and rs7803992.

***LOXL1* haplotype**	**Cases**	**Control**	***CNTNAP2* SNP**	**Cases MAF**	**Control MAF**	**p-value**
T-G carriers	103	52	rs1404699	0.413	0.308	0.072
			rs7803992	0.398	0.298	0.084
Non T-G carriers	5	147	rs1404699	0.400	0.306	0.53
			rs7803992	0.300	0.245	0.69

*LOXL1*; lysyl oxidase–like 1, MAF; minor allele frequency.

## Discussion

### Association between *CNTNAP2* and XFS

We compared the findings of Krumbiegel and colleagues [26] to that obtained from our Japanese cohorts. We found that two SNPs in *CNTNAP2* were strongly associated with XFS. In an earlier study [26], the frequencies of rs2107856 and rs2141388 SNPs in *CNTNAP2* were confirmed in an independent German cohort but not in the Italian cohort. Although neither the rs2107856 or rs2141388 SNPs was significant in our study, rs1404699 and nearby rs7803992 were statistically significant between the XFS group and the control group. Thus, it is possible that *CNTNAP2* could be associated with XFS. Like other susceptible variants of a complex disease, the OR in the earlier study was modest at about 1.4. In our study, the highest OR was 1.86 for rs7803992. This difference can be explained by racial differences and heterogeneities. Because the number of XFG patients was small, it seemed that the statistical power was weak.

### No association between *CNTNAP2* and *LOXL1* in Japanese

Because a strong association of variants in *LOXL1* in XFS has been reported [10], we compared the allele frequencies at *CNTNAP2* locus based on the presence of the identified Japanese *LOXL1* common risk haplotype T-G. We found no significant association to allele T of the rs1404699 and rs7803992 SNPs of *CNTNAP2* in carriers of *LOXL1* the risk T-G haplotype (Table 4), and also in non-risk haplotypes. These findings suggest that there is no association between *CNTNAP2* and *LOXL1* in the Japanese. This would then mean that a *LOXL1*-independent mechanism is involved in *CNTNAP2* function.

In a molecular genetic study, the most promising loci at 18q12.1–21.33 and 2q, 17p, and 19q have been proposed to be the susceptible loci in a Finnish population in an autosomal dominant mode of inheritance [31]. In a microarray study, 23 genes with different expression patterns in the anterior segment tissues of eyes with XFS have recently been reported [32]. This strongly suggests that an unidentified gene or environmental factors independent of the *LOXL1* gene strongly influence the phenotypic expression of the XFS.

### *CNTNAP2* function and molecular genetics

CNTNAP2 is a single-pass transmembrane protein with multiple protein-interaction motifs typical of the neurexins, e.g., epidermal growth factor repeats, laminin globular domains, and F5/8-type C domain, and a putative PDZ-binding site. Poliak et al. [33] reported that CNTNAP2 is necessary to maintain the potassium channels at the juxtaparanodal region in myelinated axons. The SNPs we selected were located in introns 9, 10, and 11 (Figure 1), while several SNPs related to autism were located in intron 2 [34] and intron 13 [35]. The cortical dysplasia-focal epilepsy syndrome is caused by a single nucleotide deletion in Exon 22. Therefore, it seems that our SNPs have nearly no correlation with neuropsychiatric disorders. The rs1404699 and rs7803992 SNPs are located in intron 9 of the *CNTNAP2* gene. Exon 9, nearby to intron 9, codes for the laminin globular domain, which contains proteins that play a wide variety of roles in cell adhesion, signaling, migration, assembly, and differentiation of cells. We suggest that alterations in membrane stabilization may contribute to the abnormal exfoliation matrix processes, which are associated with cell-surface irregularities, basement membrane destruction and degenerative alterations.

### Conclusions

Identification of XFS-associated SNPs that will allow early detection of an increase in the IOP, or even before an elevation of IOP, would be desirable. Our findings showed that variants of *CNTNAP2* rs1404699 and rs7803992 are significantly associated with XFS in the Japanese population. More studies of the functions and genotype-phenotype correlation of *CNTNAP2* are required to determine the pathophysiology of XFS. In addition, further studies searching for secondary genetic and environmental factors that contribute to XFS is required to gain better understanding of the complex etiology of XFS.
